# Finding the optimal tube current and iterative reconstruction strength in liver imaging; two needles in one haystack

**DOI:** 10.1371/journal.pone.0266194

**Published:** 2022-04-07

**Authors:** Bibi Martens, Joris G. A. Bosschee, Sander M. J. Van Kuijk, Cécile R. L. P. N. Jeukens, Maikel T. H. Brauer, Joachim E. Wildberger, Casper Mihl

**Affiliations:** 1 Department of Radiology and Nuclear Medicine, Maastricht University Medical Center, Maastricht, The Netherlands; 2 CARIM School for Cardiovascular Diseases, Maastricht University, Maastricht, The Netherlands; 3 Maastricht University, Maastricht, The Netherlands; 4 Department of Clinical Epidemiology and Medical Technology Assessment, Maastricht University Medical Center, Maastricht, The Netherlands; Polytechnic University of Marche, ITALY

## Abstract

**Objectives:**

The aim of the study was to find the lowest possible tube current and the optimal iterative reconstruction (IR) strength in abdominal imaging.

**Material and methods:**

Reconstruction software was used to insert noise, simulating the use of a lower tube current. A semi-anthropomorphic abdominal phantom (Quality Assurance in Radiology and Medicine, QSA-543, Moehrendorf, Germany) was used to validate the performance of the ReconCT software (S1 Appendix). Thirty abdominal CT scans performed with a standard protocol (120 kV_ref_, 150 mAs_ref_) scanned at 90 kV, with dedicated contrast media (CM) injection software were selected. There were no other in- or exclusion criteria. The software was used to insert noise as if the scans were performed with 90, 80, 70 and 60% of the full dose. Consequently, the different scans were reconstructed with filtered back projection (FBP) and IR strength 2, 3 and 4. Both objective (e.g. Hounsfield units [HU], signal to noise ratio [SNR] and contrast to noise ratio [CNR]) and subjective image quality were evaluated. In addition, lesion detection was graded by two radiologists in consensus in another 30 scans (identical scan protocol) with various liver lesions, reconstructed with IR 3, 4 and 5.

**Results:**

A tube current of 60% still led to diagnostic objective image quality (e.g. SNR and CNR) when IR strength 3 or 4 were used. IR strength 4 was preferred for lesion detection. The subjective image quality was rated highest for the scans performed at 90% with IR 4.

**Conclusion:**

A tube current reduction of 10–40% is possible in case IR 4 is used, leading to the highest image quality (10%) or still diagnostic image quality (40%), shown by a pairwise comparison in the same patients.

## Introduction

Computed tomography (CT) is used on a daily basis in abdominal imaging to detect and evaluate a variety of pathologies [1, 2]. While its clinical importance and benefits are undisputed, CT uses ionizing radiation and radiation exposure may lead to an increase in the lifetime attributable cancer risk [3, 4]. Radiation exposure should therefore be kept “as low as reasonably achievable” according to the ALARA principle [5]. Conversely, excessive radiation dose reduction can lead to non-diagnostic scans. From a radiation safety point of view this is the worst-case scenario as no medical benefit was gained from the radiation exposure and a retake will lead in summary to an increase of the total radiation exposure. This is indicative of the delicate balance between radiation dose and image quality.

Several dose reduction techniques are applied in daily clinical practice, such as automated tube current modulation (ATCM), automated tube voltage selection (ATVS) and iterative reconstruction (IR) techniques [6–15]. ATCM and ATVS optimize radiation dose by optimizing the tube current and tube voltage during the acquisition to reach diagnostic image quality for each individual patient [8, 10]. IR techniques are used during reconstruction of the scans to further decrease image noise, without compromising image quality [9]. Based on the same raw data IR techniques result in a decrease in image noise, by repetitive calculation steps during the reconstruction. The repetition is stopped when a predefined number of cycles is reached, or when the difference between two IR steps becomes smaller than a predefined amount [16]. The achievable decrease in image noise, which is related to the IR strength, can be relinquished in favour of a radiation dose reduction [17]. Previous studies have shown IR techniques to be superior to filtered back projection (FBP). Although, while Hardie et al. showed a reader preference for low to intermediate IR strengths, Choy et al. demonstrated a preference for images reconstructed with IR strength 4 or 5 [18, 19]. Noise decreases with an increased IR strength, but at the same time, the texture of the noise changes, possibly negatively influencing image quality [20]. Therefore, in daily clinical practice, different IR strengths are used, which may also depend on the scan phase (e.g. arterial, portal venous or delayed phase) [18, 21–23].

Comparing image quality between CT scans of different patients is challenging, because differences in patient-related factors (e.g. height, weight, liver morphology and cardiac function) affect image quality [6, 15]. Ideally, if one patient could be scanned several times, a reliable search for the most optimal tube current and tube voltage could be performed. Reconstruction software allows to reconstruct multiple lower tube current scans from a single raw data set. Therefore, it provides the opportunity for pairwise comparison of identical patients without the need for repetitive scanning. This software can aid in finding the optimal reference tube current and help in further decreasing radiation exposure. Previous studies showed that using dedicated post-processing software (ReconCT) for optimization a potential dose reduction of 41 to 84% was possible in CT angiography (CTA) of various vascular structures in head and neck without compromising diagnostic image quality [24, 25]. The pairwise comparison stipulates the opportunity to evaluate whether a dose reduction still leads to diagnostic image quality and lesion detection in abdominal imaging. The latter being one of the most important parameters, as an increased risk of missing lesions is an unfavourable outcome. In a previous study, signal to noise ratio (SNR) and contrast to noise ratio (CNR) values of respectively 8.8 +/- 1.8 and 5.5 +/- 2.1 led to a good to excellent subjective image quality in 93.7% of the patients [26]. Therefore, it is safe to assume that a SNR above 8.0 and a CNR above 5.0 are considered diagnostic.

The aim of this study was to assess both the optimal IR strength and the lowest possible tube current in abdominal CT imaging while maintaining diagnostic image quality with the use of ReconCT software.

## Materials and methods

### Ethical considerations

This study was provided a waiver of written informed consent by the local ethical committee and institutional review board as retrospective data were analysed anonymously (ref METC 2017–0250).

### Study design

ReconCT software (version 13.0.0.1, prototype software, Siemens Healthineers, Forchheim, Germany) was used to reconstruct raw image data at lower tube currents and different IR strengths to simulate radiation dose reduction and accompanying image quality, i.e. an increase in noise. The raw CT image data were exported directly from the CT-scanner. All scans were performed on a 3^th^ generation dual-source CT (DSCT) scanner (Somatom Force, Siemens Healthineers, Forchheim, Germany). Validation of the software has been published elsewhere [24, 25]. Nevertheless, a quality assurance has been performed in a phantom study (S1 Appendix).

Thirty abdominal scans were retrospectively selected, based on the used scan and contrast media (CM) injection protocol. Scans performed in portal venous phase at 90 kV (120 kV_ref_ and 150 mAs_ref_), with a CM injection protocol of 0.4 g I/kg were eligible for inclusion. The presence or absence of liver lesions was not evaluated. Scans were reconstructed multiple times with lower mAs percentages and IR strengths. Both objective and subjective image quality were evaluated as explained below. In addition, 30 abdominal scans all containing a diversity of liver lesions, were retrospectively selected to determine the optimal IR strength for lesion detection based on the results of the previous steps.

### Patient study

Thirty abdominal scans of unique patients acquired in portal venous phase were included between September 2019 and February 2020. Inclusion criteria were scans in which ATCM (CareDose 4D; Siemens) and ATVS (CARE kV; Siemens) techniques were used, with a reference tube voltage and tube current of respectively 120 kV_ref_ and 150 mAs_ref_, with a slice collimation of 192 x 0.6 mm and gantry rotation time 0.5 seconds. Only scans acquired at 90 kV in which a dosing factor of 0.4 g I/kg CM was used, were included to ensure a homogeneous database. Dedicated CM injection software was used (P3T; Bayer Healthcare, Berlin, Germany), which calculates CM volume and flow rate, based on the linear relationship between body weight and injection duration [15]. A history of liver disease or surgery was not a reason for exclusion. General exclusion criteria for a contrast-enhanced abdominal CT were applied (e.g. pregnancy, renal insufficiency [estimated glomerular filtration rate < 30 mL/min per 1.73 m^2^] and iodine allergy).

The raw data of the selected scans were transferred to the ReconCT workstation, where the scans reconstructed with lower tube currents were simulated. As ATCM and ATVS techniques were used, the mAs_eff_ differed between patients, this mAs_eff_ will hereinafter be referred to as the initial value. Data were reconstructed with tube current of 90%, 80%, 70% and 60% of the initial value. Based on the phantom study (S1 Appendix), reconstructions with a tube current below 60% were expected to be of non-diagnostic image quality, and therefore, these data were not simulated. In addition, all scans were reconstructed with FBP and IR strengths 2, 3 and 4 (Advanced Modeled Iterative Reconstruction [ADMIRE], Siemens Healthineers, Forchheim, Germany), kernel Br40. This resulted in a total of 510 CT series (17 reconstructions for each patient).

Patients’ weight was asked prior to the CT scan and together with patient’ sex, age and radiation dose information (e.g. mean effective mAs [mAs_eff_], CT dose index [CTDI_vol_, in mGy] and dose length product [DLP, in mGy∙m]) collected from the PACS workstation (IMPAX version 6.6.1.5003, AGFA HealthCare N.V., Mortsel, Belgium). The CM volume (in ml), total iodine load (TIL, g I), flow rate and iodine delivery rate (IDR, in g I/s) were monitored with a dedicated data acquisition program (Certega^™^ Informatics Solution; Bayer).

#### Image analysis

Data were transferred to the radiology workstation (SyngoVia^™^, VB30; Siemens Healthineers, Erlangen, Germany). In all reconstructions, the mean Hounsfield Units (HU) and standard deviation (SD) were measured by placing the largest possible region of interest (ROI) in three different liver segments (area ≥ 1 cm^2^), preferably segments 2, 5 and 8 (according to the Couinaud distribution), not containing vessels, biliary ducts or regional anomalies (e.g. cysts, metastasis or changes related to surgery) [27]. The signal to noise ratio (SNR) was calculated by dividing the mean HU of the liver by its SD. The difference between the mean liver HU and the attenuation of the left paraspinal muscle, divided by the SD of the paraspinal muscle resulted in the contrast to noise ratio (CNR).

#### Subjective image quality

Two radiologists (B.M. and C.M.) with respectively 4- and 9-years’ experience in abdominal imaging rated all scans in consensus on diagnostic screens, while being blinded to the simulated tube current and reconstruction method used. All scans were presented in a random order. The radiologists were allowed to adjust window levels. The overall image quality and lesion detection capability were separately rated on a 5-point Likert scale (1 very poor, 2 = poor, 3 = moderate, 4 = good, 5 = excellent) [15, 28]. In search for an optimal image quality, scans rated as “good” or “excellent” were considered of diagnostic image quality. The simulated scans with the highest percentage of scans with good or excellent image quality, were rated best.

### Liver lesions

In addition to the previous patient study, 30 abdominal scans containing a diversity of liver lesions (e.g. non-specific, benign or malignant) were collected between June and August 2020. Scans were used for the evaluation of lesion detection as the presence of actual lesions, makes it easier and more reliable to evaluate this parameter. Scans were eligible for inclusion when the same scan and CM injection protocol as in the patient study was used. IR strength 3, 4 and 5 were reconstructed on the scanner, based on the results of the first patient study. Two radiologists (B.M. and C.M.) evaluated in consensus which IR strength resulted in the best liver lesions detectability. The readers had to choose the preferred strength out of the three reconstructed IR strengths. The IR strength rated most often as best for lesion detection, was declared the favoured strength.

### Statistical analysis

Summaries of categorical variables were expressed as absolute numbers with percentages and continuous variables as mean ± SD. A linear mixed-effects model was used to account for the fact that of each patient 17 reconstructions were made and hence, data were correlated. The effective mAs was added to the model as a covariate, with HU as a dependent variable. The generalized mixed-effects model with binomial link function was used to investigate if there was an association between the IR strength and the dichotomized subjective image quality (diagnostic image quality and lesion detection). Results of the generalized linear mixed-effects model were expressed as odds ratio (OR) and 95% confidence interval (CI). Statistical software (SPSS, version 26.0; IBM Corp, New York, NY) was used for the data analysis.

## Results

### Patient study

Baseline characteristics of the population are depicted in Table 1. Mean mAs_eff_, CTDI_vol_, DLP, CM volume, TIL, flow rate and IDR are shown in Table 2. Data from one patient were excluded, as a higher dosing factor (in g I/kg) was used.

**Table 1:** Baseline characteristics.

*Parameters*	*N = 29*
*Age (years)*	*64.9 ± 14.2*
*Sex (% male)*	*18 (62*.*1%)*
*Body weight (kg)*	*72.2 ± 9.9*
*Height (m)*	*1.73 ± 0.1*
*BMI (kg/m* ^*-2*^ *)*	*24.2 ± 2.4*
Liver lesions present (% yes / mean number of lesions)	62% / 5.4 ± 12.3
Type of lesion	
Metastasis (N)	2
Cysts (N)	12
Postoperative (N)	3
Hemangioma (N)	1
Mean maximum lesion size (mm)	15.0 ± 8.1
Mean minimum lesion size (mm)	6.1 ± 4.0

BMI indicates body mass index.

**Table 2:** Radiation dose and injection parameters.

*Parameters*	*N = 29*
*Mean mAs*_*eff*_	*212.0 ± 27.9*
*CTDI*_*vol*_ *(mGy)*	*6.1 ± 0.8*
*DLP (mGy∙cm)*	*291.4 ±43.8*
*CM volume (ml)*	*95.8 ±13.1*
*TIL (g)*	*28.8 ± 3.9*
*Flow rate (ml/s)*	*2.9 ± 0.6*
*IDR (g I/s)*	*0.96 ± 0.1*
*Dosing factor (g I/kg)*	*0*.*4*

mAs_eff_ indicates effective tube current; CTDI_vol_, CT dose index_vol_; DLP, dose length product; CM, contrast media; TIL, total iodine load; IDR, iodine delivery rate.

**Fig 1:**
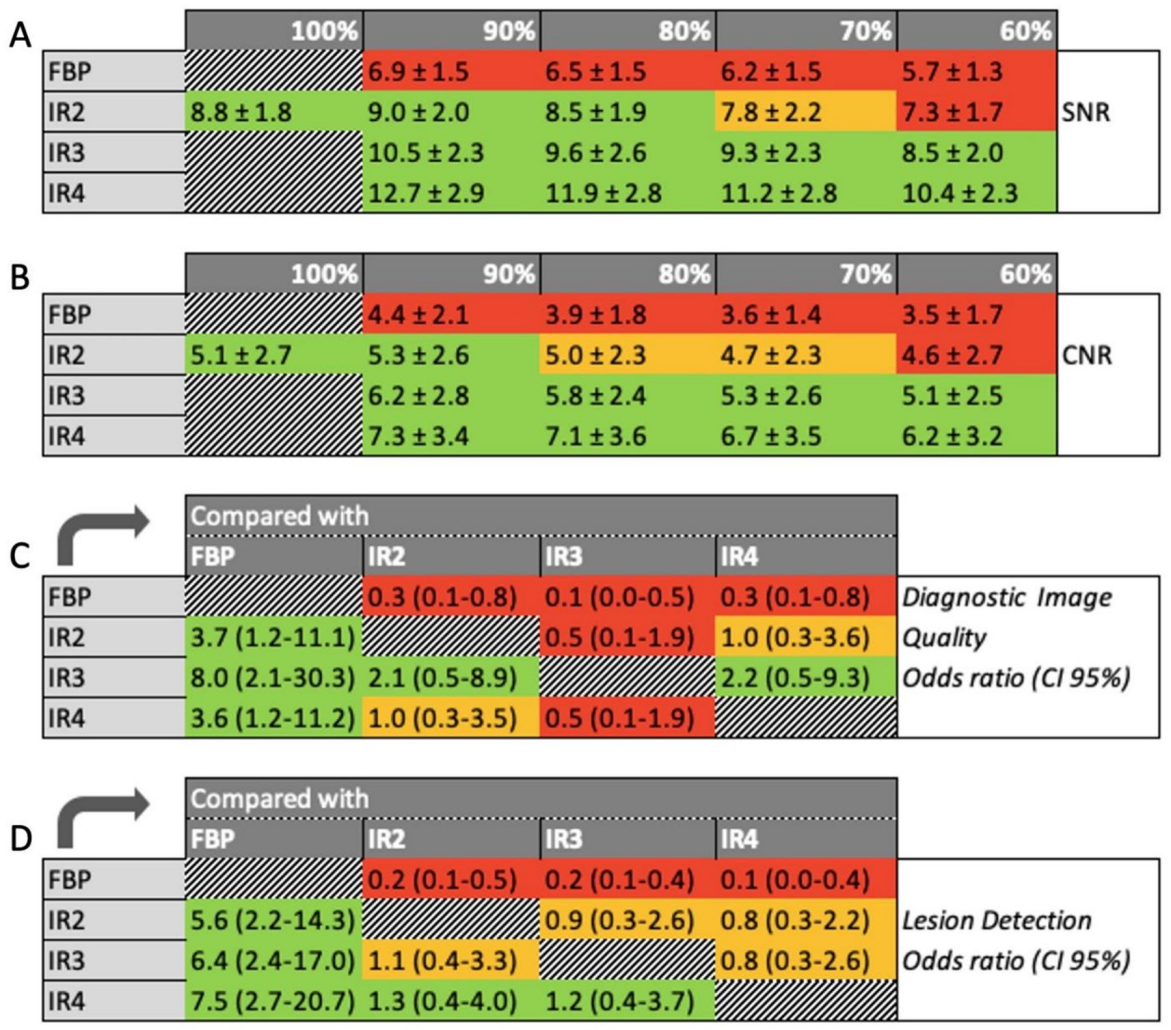
A signal to noise ratio (SNR) of 8.0 (A) and contrast to noise ratio (CNR) of 5.0 (B) were considered diagnostic. A and B show the corresponding SNR and CNR for each combination of iterative reconstruction (IR) strength and percentage of the initial value of the tube current. In green the combinations leading to diagnostic objective image quality. In part C and D, the odds ratios of the overall diagnostic image quality (C) and the lesion detection capability (D) are set out. Filtered back projection (FBP) and IR strengths on the left are compared to the reconstruction methods on the x-axis. For example, the odds that IR 4 results in a better lesion detection than IR 3 is 1.2, with a confidence interval (CI) of 0.4–3.7.

To find the optimal tube current and IR strength, SNR and CNR were evaluated (Fig 1). Fig 1a and 1b show in green which percentage in mAs reduction still leads to a diagnostic SNR and CNR. In case IR strength 3 or 4 is used, a mAs of 60% still results in diagnostic SNR and CNR. Fig 1c depicts the overall image quality with each reconstruction strength. The odds that IR strength 3 results in a diagnostic scan was eight times higher than that of FBP and more than two times higher than strength 2 and 4. The odds that IR 4 results in a better lesion detection was 7.5 times higher than that of FBP and respectively 1.3 and 1.2 times higher than that of IR 2 and IR 3 (Fig 1d).

A mAs of 60% with the use of IR strength 3 or 4, still leads to diagnostic objective image quality. The overall subjective diagnostic image quality was highest for IR 3. IR 4 was graded best for lesion detection.

The percentage of scans considered of diagnostic image quality (rated as of good or excellent diagnostic image quality) was highest (89.7%) for the scan at 100% with IR 2. With IR 3 at 90, 80, 70 and 60% respectively 79.3, 69.0, 65.5 and 48.3% of the scans was rated diagnostic. Diagnostic image quality was reached in 82.8, 72.4, 79.3 and 55.2%, respectively at IR 4. Regarding lesion detection, the percentage of diagnostic scans was 79.3% at 100% with IR 2, while at IR 3 the percentages at 90, 80, 70 and 60% were respectively 72.4, 62.1, 55.2 and 37.9%. At IR 4 the percentage of scans rated as excellent or good was 86.2, 69.0, 69.0 and 55.2% respectively (Table 3).

**Table 3:** Percentage of scans rated as of good or excellent diagnostic image quality, and rated as good or excellent lesion detection capability, set out per reconstruction method.

*Reconstruction percentage*	*FBP*		*IR 2*		*IR 3*		*IR 4*	
	Overall IQ	Lesion detection	Overall IQ	Lesion detection	Overall IQ	Lesion detection	Overall IQ	Lesion detection
*100%*			89.7	*79*.*3*				
*90%*	44.8	31.0	75.9	65.5	*79*.*3*	72.4	*82*.*8*	86.2
*80%*	31.0	24.1	69.0	58.6	*69*.*0*	62.1	*72*.*4*	69.0
*70%*	34.5	27.6	65.5	58.6	*65*.*5*	55.2	*79*.*3*	69.0
*60%*	20.7	10.3	34.5	31.0	*48*.*3*	37.9	*55*.*2*	55.2

### Liver lesions

**Fig 2:**
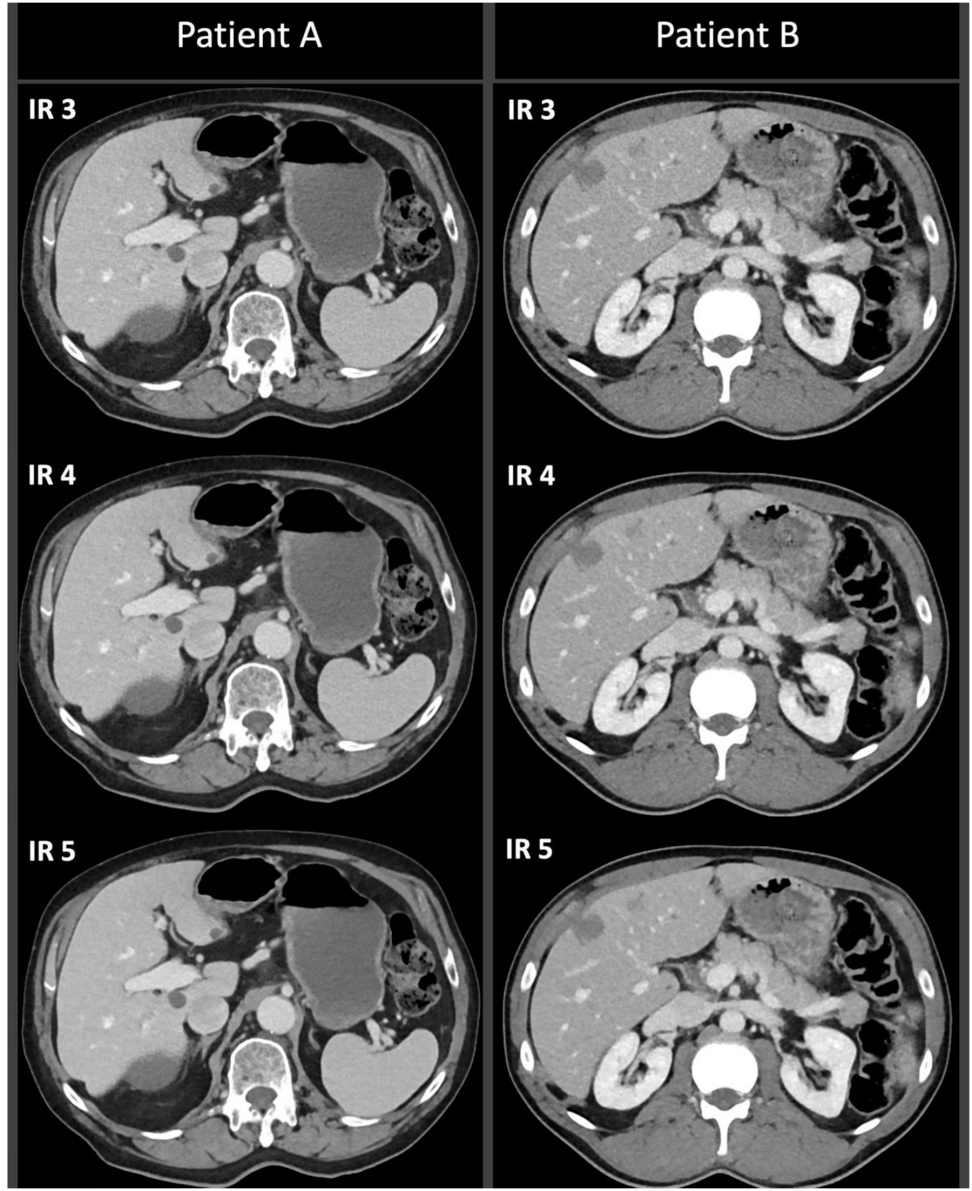
Abdominal scan of an 86-year-old patient (Patient A) in the follow-up for a urothelial cell carcinoma, who has multiple cysts in the liver parenchyma. In addition, a scan of a 47-year-old male (Patient B) in the follow-up for hepatic metastasis of colorectal cancer. Both scans are reconstructed with IR strength 3, 4 and 5. The scans reconstructed with IR 4 were rated in consensus to have the best lesion detection capability.

This second patient population confirmed the preference for IR strength 4 regarding lesion detection. In twenty-five cases IR strength 4 was most appreciated, while IR 3 was valued highest in 4 cases and IR 5 in only one case. Examples of two cases in which IR 4 was preferred are depicted in Fig 2.

## Discussion

The aim of the study was to find the optimal IR strength and the lowest possible (reference) tube current that could be used in abdominal CT imaging, without compromising objective and subjective image quality. In accordance with the literature, IR techniques outperformed FBP [18, 19, 22]. When IR techniques are used, the mAs_ref_ can be reduced without compromising objective image quality. The results indicate that with IR strength 3 or 4, reductions of up to 40% still produce a diagnostic SNR and CNR. Scans performed with IR 4 at 90% tube current, led to a slightly higher lesion detection capability compared to the full dose at IR strength 2. Therefore, it can be concluded that the mAs_ref_ in abdominal imaging can be safely reduced by 10–40%, in case IR strength 4 is used on this particular scanner, showed by pairwise comparison. Ten percent reduction at IR 4 leads to the highest image quality, while a reduction of 40% at IR strength 4 still results in diagnostic image quality.

For the first patient study, only scans with IR strength 2 to 4 were reconstructed. IR strength 1 and 5 were not reconstructed. From experience, IR 1 was expected to result in very noisy images and IR 5 in images appearing very smoothened. IR 4 turned out to result in subjectively the best lesion detection capability. Subsequently, the second study was performed, in which IR 5 was incorporated in addition to IR strength 3 and 4 to not rule out possible superiority of IR strength 5.

A number of studies have evaluated the possibility to reduce radiation dose in abdominal imaging [23, 29–33]. To the best of our knowledge, this is the first study comparing different radiation doses and IR strengths in abdominal imaging, within the same patient by using reconstruction software. The study set up can be used to investigate the optimal tube current and IR algorithm for each anatomical region, scan indication, vendor and specific scanner.

Our study evaluated both objective and subjective image quality. For the latter, as the name already implies, it is subjective and some readers might prefer more noise for a particular scan indication, while others prefer more smoothened scans [17]. Establishing the objective image quality with SNR and CNR seems rather straightforward. Although, when searching for reliable thresholds, a wide variety of values is found in the literature, all presumed to be of diagnostic image quality, and no clear cut-off values have been established [33–37]. In addition, previous literature states that both SNR and CNR might not encompass the complete appreciation of image quality [38, 39]. The present study sets out the discrepancy between objective and subjective image quality. According to the SNR and CNR values a radiation dose reduction of 40% was possible, while looking at the subjective parameters only a smaller tube current reduction of 10–40% seemed feasible. This indicates the struggle to be able to safely declare that CT scans are of diagnostic image quality. Future research could focus on determining new, more universal objective parameters to reliably, generalizably and consequently assess image quality. Such parameters would make it possible to establish with a higher degree of certainty if image quality is diagnostic and if all the different developed radiation dose reduction algorithms and reconstructions results in diagnostic image quality for diagnostic purposes.

### Limitations

The current study is a single-center study with a rather small patient sample. In addition, the golden standard for lesion detection and characterization is autopsy, which was not performed. The baseline protocol for abdominal imaging chosen in present study was the scan and CM injection protocol as used in daily clinical practice. This assumes that this baseline scan protocol is considered to be of good–maybe even too good–image quality, while this protocol might potentially have benefitted from a (small) increase in dose. In addition, only scans performed in portal venous phase were included, whereby the presence or absence of liver lesions was not a selection criterion. Therefore, present study lacks generalizability to other scan phases and protocols, which leaves room for future studies. Lastly, radiation dose reduction and IR strengths were only studied on a CT scanner from one vendor, which limits generalizability of the outcome. As the software is vendor specific and raw data based it is therefore not applicable to scanners form other vendors.

## Conclusion

IR strength 4 leads to the best subjective image quality in abdominal CT imaging and gives the opportunity to reduce the tube current by 10 to 40% without compromising both objective as well as subjective image quality as shown by pairwise comparison in the same patients with the use of ReconCT software.

## Supporting information

S1 Appendix Phantom study for validating ReconCT software. (DOCX)
