# Supplementary material for: Finding the optimal tube current and iterative reconstruction strength in liver imaging; two needles in one haystack
Source: PLoS One. 2022 Apr 7;17(4):e0266194. doi: 10.1371/journal.pone.0266194 (PMC8989341; doi:10.1371/journal.pone.0266194)
Supplement: S1 Appendix — (DOCX) [file pone.0266194.s001.docx]

**S1 Appendix – Phantom study for validating ReconCT software**

**Quality Assurance**

**Material and Methods**

To validate the performance of the ReconCT software, a phantom study was performed using a semi-anthropomorphic abdominal phantom (Quality Assurance in Radiology and Medicine, QSA-543, Moehrendorf, Germany), not containing (liver) lesions. Automated tube current modulation (ATCM) (CareDose 4D; Siemens) and automated tube voltage selection (ATVS) (CARE kV; Siemens) were both turned off. Acquisition parameters were: a tube voltage of 90 kV, effective tube current of 295 mAs, slice collimation 192 x 0.6 mm and gantry rotation time 0.5 seconds. Subsequently, scans with a reduced tube current in steps of 10 % dose reduction were acquired at: 266, 236, 207, 177, 148, 118, 89, 59 and 30 mAs (figure 1). Every scan was reconstructed with both filtered back projection (FBP) and iterative reconstruction (IR) strength 1 to 5 (Advanced Modeled Iterative Reconstruction [ADMIRE], Siemens Healthineers, Forchheim, Germany). Image reconstruction was performed with 3 mm slice thickness and an increment of 2 mm.

The raw data of the scan acquired with 295 mAs was used as input for the ReconCT software. Simulations of 90 %, 80 %, 70 %, 60 %, 50 %, 40 %, 30 %, 20 % and 10 % of the initial mAs-value were created from the dataset. In addition, the software was used to reconstruct scans with FBP and IR strength 1-5 (figure 1).

*Image analysis*

All scans were evaluated on a dedicated radiology workstation (SyngoVia^TM^, VA30; Siemens Healthineers, Erlangen, Germany). Objective image quality of both the measured and simulated phantom data was evaluated by measuring the mean Hounsfield Units (HU) and the standard deviation (SD) in the liver and the background using a region of interests (ROI) as large as possible (> 2 cm^2^). The ROI was placed on one of the middle slices, the same slice was used for each scan. The signal to noise ratio (SNR) was calculated by dividing the mean HU of the liver by its SD, while the contrast to noise ratio (CNR) was calculated by dividing the difference of the mean liver HU and mean background HU by the background SD (26). In case the SNR and CNR of the measured data was comparable or better than the simulated data, the software was considered validated. This criterium ensures that the results of the simulated data can be safely translated to real life (measured) data.

**Results**

Figure 2 shows the SNR and CNR in both the measured and simulated data for all reconstructed percentages of the tube current. As expected, the SNR and CNR decrease with decreasing percentage of the tube current. The simulated data did not outperform the measured data.


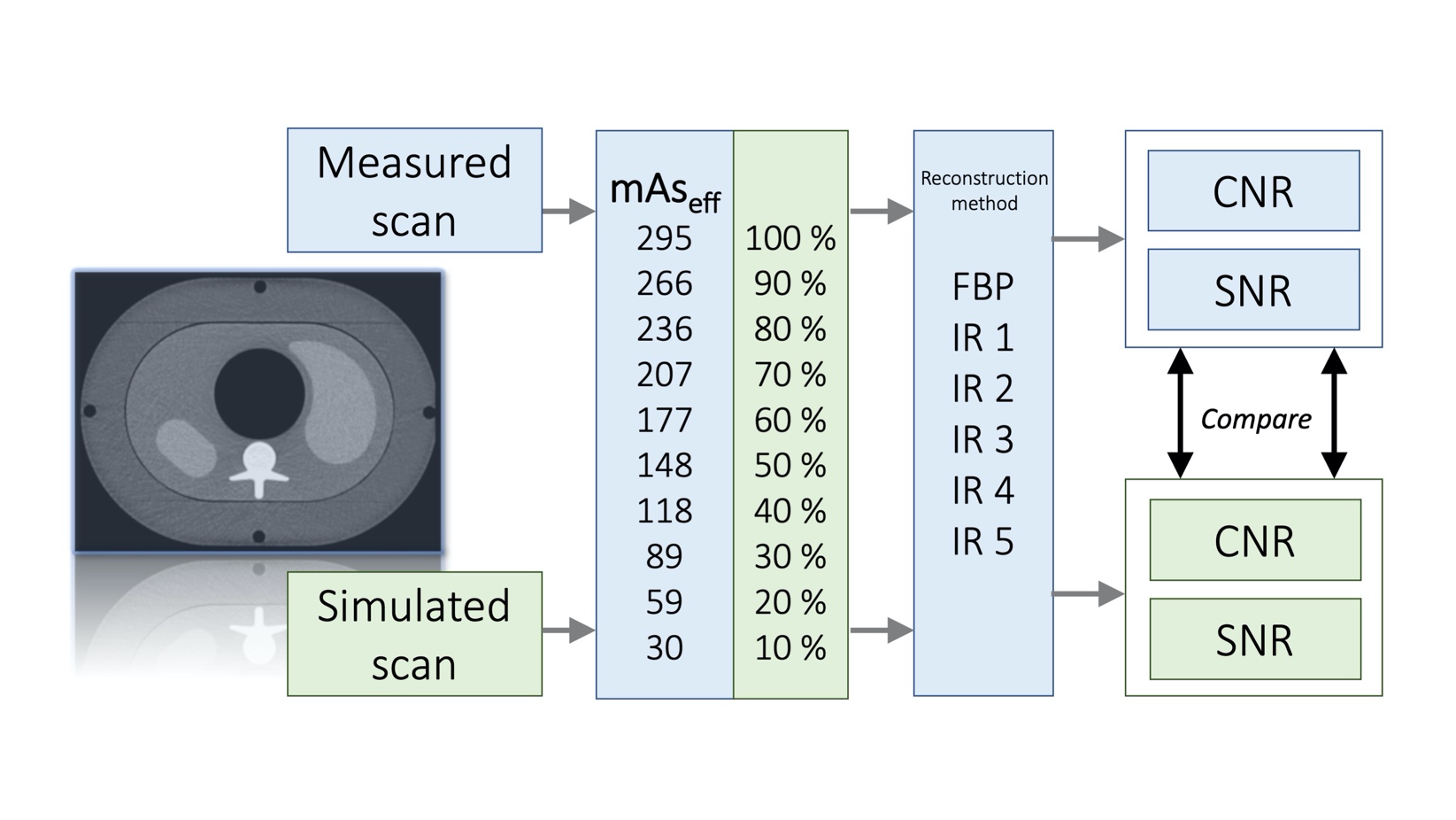


**Figure appendix-1.** Phantom study protocol in which a semi-anthropomorphic abdominal phantom (Quality Assurance in Radiology and Medicine Moehrendorf, Germany) was scanned with the above-mentioned effective mAs (mAs_eff_) and reconstructed with the different reconstruction methods and strengths (FBP, filtered back projection; IR, iterative reconstruction), called the measured scan. In addition, the scan performed with 295 mAs_eff_ was reconstructed with the ReconCT software (Simulated scan), with the different mAs_eff_ and reconstruction settings as mentioned above. Lastly, the signal to noise ratio (SNR) and contrast to noise ratio (CNR) were compared between the measured and the simulated phantom scan.

**
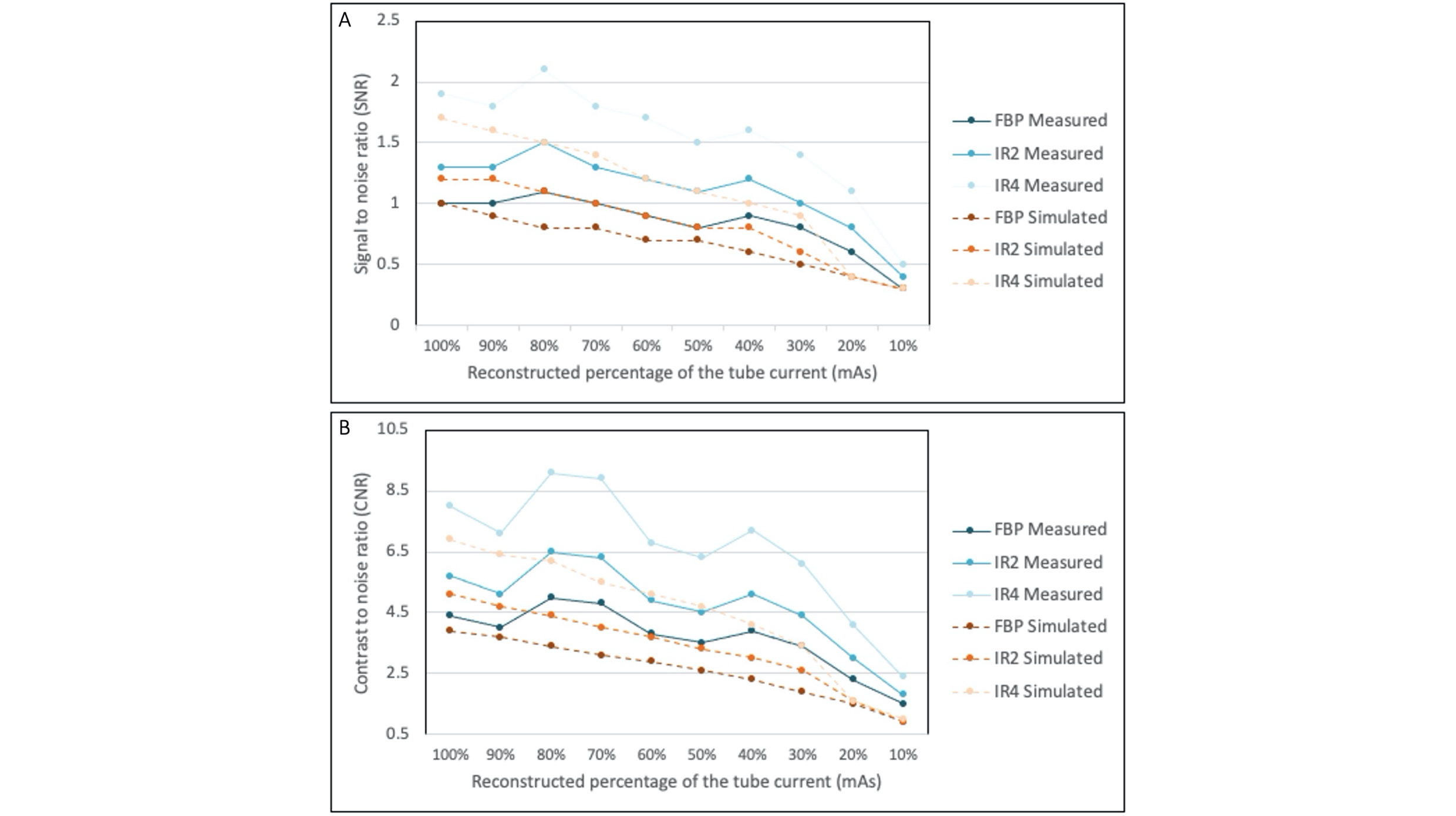
Figure appendix-2.** The measured and simulated mean signal to noise ratio (SNR) in A and the contrast to noise ratio (CNR) in B in a semi-anthropomorphic abdominal phantom (Quality Assurance in Radiology and Medicine, Moehrendorf, Germany). Both the SNR and CNR of the simulated data do not outperform the measured data in all instances.
